# The effect of spaced E-Learning on knowledge of basic life support and satisfaction of nursing students: a quasi-experimental study

**DOI:** 10.1186/s12909-024-05533-9

**Published:** 2024-05-15

**Authors:** Fataneh Ranjbar, Hamid Sharif-Nia, Maryam Shiri, Pardis Rahmatpour

**Affiliations:** 1 Student Research Committee, Alborz University of Medical Sciences, Karaj, Iran; 2 Education Development Center, Mazandaran University of Medical Sciences, Sari, Iran; 3 Department of Nursing, Amol Faculty of Nursing and Midwifery, Mazandaran University of Medical Sciences, Sari, Iran; 4 Department of Medical Education, Alborz University of Medical Sciences, Karaj, Iran; 5 School of Nursing, Alborz University of Medical Sciences, Karaj, Iran

**Keywords:** Online education, Cardiopulmonary resuscitation, Student satisfaction, Spaced learning

## Abstract

**Aim:**

Cardiopulmonary resuscitation (CPR) training is essential for all students, especially nursing students. One of the educational approaches to creating long-term learning in inclusive is spaced learning. Spaced learning consists of three or more training sessions in which information is presented over time and at intervals. The present study was conducted to investigate the effect of basic life support (BLS) training through spaced E-learning on the knowledge and satisfaction of nursing students.

**Methods:**

In this quasi-experimental study with two groups, 106 undergraduate nursing students of Alborz University of Medical Sciences in Iran participated. The control group (*n* = 47) received BLS training with massed E-learning in one three-hour session, and the intervention group (*n* = 59) received spaced E-learning in three one-hour sessions. An electronic questionnaire including demographic information and a pre-test of BLS knowledge were sent to both groups. Also, immediately after receiving the training, two weeks later and one month later, they completed a post-test of BLS knowledge. Students were asked to indicate their level of satisfaction with the provided education by completing the SLS-OLE.

**Results:**

The post-test scores immediately after, two weeks later, and one month later of the intervention group were higher than the control group. The results of repeated measurement ANOVA showed that the score changes of knowledge are significant over time (*p* < 0.001), the number of sessions (*p* < 0.001), and the interactive effect of them (*p* < 0.001). There was no statistically significant difference in the level of satisfaction with education in both groups.

**Conclusion:**

Based on the results, BLS training in both groups increased the knowledge of BLS. however, the increase in knowledge and its retention was higher in the intervention group that received the training in spaced learning.

## Introduction

Cardiopulmonary resuscitation (CPR) stands as an indispensable skill for nurses and nursing students, wielding a direct impact on patient survival rates. The prompt initiation of basic cardiopulmonary resuscitation (BLS) remains pivotal in determining client survival [1, 2].

With nursing students being a common presence in healthcare institutions, one would expect them to possess ample and up-to-date knowledge and skills [3]. Although BLS is introduced during high school education, reinforcing this training at the university level becomes essential due to the unique demands of nursing. In Iran, BLS is currently integrated into the curriculum during the 6th semester of undergraduate nursing programs. However, nursing students engage with patients from the second semester onwards, both inside and outside the hospital setting. Consequently, CPR knowledge and skills assume a paramount role in their education [4].

Recent studies present a divergence in opinions regarding the most effective method for CPR training. Some have highlighted the efficacy of virtual reality simulation, educational videos, and gamification in teaching BLS [5–9]. Students exposed to CPR training across multiple sessions, incorporating diverse educational technologies, exhibit increased motivation and self-confidence in executing this life-saving procedure [10]. They also demonstrate superior self-efficacy and long-term retention of CPR knowledge and skills [11]. Repetition emerges as a crucial element in minimizing errors and boosting learners’ self-assurance, with the frequency of CPR training sessions correlating with students’ self-confidence and willingness to learn [12, 13].

Despite the numerous methods available, CPR training continues to be predominantly delivered in the traditional “mass education” format, where all content is typically covered in a single day. However, contemporary research underscores the effectiveness of the Spaced Learning method, underpinned by the “Encoding Variability Theory”.

### The encoding variability theory

Research has shed light on the diminishing efficacy of extended, monotonous lectures exceeding 50 min, leading to students’ boredom, waning concentration, and dissatisfaction [14]. The “Spacing Effect” underscores that disseminating content at intervals within a specified timeframe yields more effective learning outcomes than presenting it all at once. The “Encoding Variability Theory” posits that varying learning conditions activate distinct areas of the brain, facilitating improved information retrieval from memory [15]. This theory underscores the importance of repeating stimuli at longer intervals, fostering effective information assimilation and retention while enhancing memory recall and information retrieval [16].

### Spaced learning

Spaced learning, an approach informed by the “Encoding Variability Theory,” champions knowledge retention by spacing out learning sessions. This method, also employed in online education, reduces students’ stress and cultivates an enjoyable learning experience. It is defined by incorporating three or more sessions that offer information over time at intervals. Spacing formats may vary in terms of timing, with some researchers implementing sessions over several days, while others structure them across hours, weeks, or months [17, 18].

This method boasts several advantages, including reducing learner distraction by introducing breaks and spacing between sessions, making it ideal for comprehending complex concepts by allowing learners to digest information before proceeding to the next segment. Additionally, each session concludes with a recap of the material covered, fortifying retention [19–21].

Several studies have explored the application of the spaced learning method in CPR training, yielding promising results. A systematic review conducted by Yeung et al. (2020), encompassing basic and advanced CPR, found that 15 out of 17 studies reported enhanced learner performance through spaced learning [22]. In a study by Oermann et al. (2020) investigating the ideal interval for CPR training, daily and weekly intervals were found to be conducive to effective learning [23]. In line with the literature, content tailored to students’ needs, comprehensive and well-designed, delivered continuously, bolsters student satisfaction [24, 25]. Given the critical importance of CPR training for nursing students and the demonstrable benefits of Spaced Learning in knowledge retention, this study endeavors to evaluate the impact of BLS Spaced E-learning on the knowledge and satisfaction of first-year undergraduate nursing students in Iran.

## Methods

### Study design

This study adopts a quasi-experimental design involving two groups, conducted among first-year undergraduate nursing students at Alborz University of Medical Sciences, Iran, 2022.

### Participants

Among 120 eligible undergraduate nursing students,106 students participated in this study, selected through a convenience sampling method. Inclusion criteria encompassed a willingness to participate, first-year nursing students with no prior CPR knowledge, no previous participation in CPR workshops, and no involvement in similar CPR research. Exclusion criteria included unwillingness to continue participation, irregular class attendance, and incomplete questionnaire responses. Invitation posters were distributed to students, and volunteers were enrolled. Eligibility criteria were verified by the researcher, and participants were randomly divided into two groups of control group (*n* = 47) and intervention group (*n* = 59). The intervention and control groups were homogeneous in terms of age, grade point average, academic semester and interest in nursing. (*p* > 0.05).

### Questionnaires

The study employed an online questionnaire comprising three forms: demographic and educational information, BLS knowledge assessment, and student satisfaction with online learning. The questionnaire was hosted on the Persian online platform (www.porsline.ir), and students were provided with a link through their Telegram groups.

Demographic and Educational Information: This form gathered data on participants’ year of birth, gender, academic semester, GPA of the last semester, and their level of interest in nursing (one question).

BLS Knowledge: This section contained 15 multiple-choice questions based on the BLS guidelines of the American Heart Association (AHA 2020), textbooks, and National Council Licensure Examination (NCLEX RN) tests. Ten faculty members in the field of medical emergency and nursing evaluated the questions for face and content validity. Professors who had the experience of teaching emergency medicine in class and internships were selected. They assessed grammar, word usage, clarity, and question relevance. Necessary revisions were made based on their feedback and CVI and CVR ratings.

Student Learning and Satisfaction in Online Learning Environments Instrument (SLS-OLE): This questionnaire consisted of 34 questions across six domains: “course structure, learner interaction, student engagement, instructor presence, student satisfaction, perceived learning,” using a seven-point Likert scale ranging from strongly disagree (1) to strongly agree (7). After obtaining permission from the scale’s developer [26], the questionnaire was translated into Persian by the researcher and underwent face and content validity evaluation by ten professors in nursing and medical education. The items’ Content Validity Index (CVI) and Content Validity Ratio (CVR) were calculated, and the items were deemed valid (CVI > 0.7, CVR > 0.62). In this study, the internal consistency of SLS-OLE was assessed using Cronbach’s alpha, resulting in values of 0.78 for course structure, 0.82 for learner interaction, 0.72 for student engagement, 0.78 for instructor presence, 0.94 for student satisfaction, and 0.88 for perceived learning.

### Research procedure

**Fig. 1:**
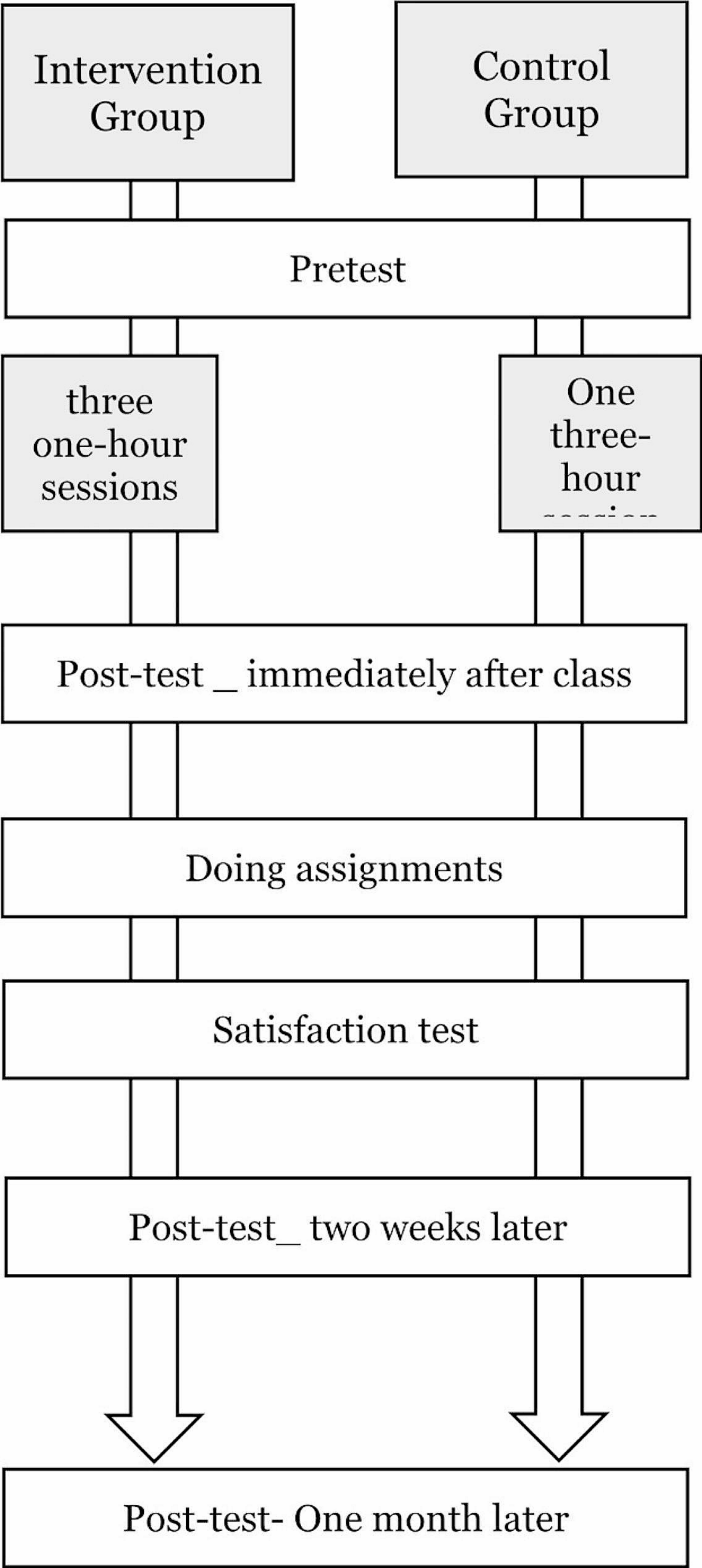
Tracking diagram of study procedure

In this study, Synchronous online teaching sessions were approximately 3 h. The control group received BLS training through massed learning in a single three-hour session, while the intervention group received spaced learning across three one-hour sessions (Fig. 1). Both groups were added on the Telegram groups. A message was sent to both groups, detailing the research objectives, confidentiality, participants’ freedom to engage or withdraw, and the research steps.

The BLS online course was taught by the researcher, a master’s student in emergency nursing with seven years of experience in the emergency field, and prior participation in CPR workshops, remaining updated on the latest AHA guidelines (BLS-AHA 2020 Guideline). The course content comprised PowerPoint presentations, videos, and images, all reviewed by two nursing professors.

The date and time for the online classes were scheduled based on student input, avoiding conflicts with their class schedules and the 2022 World Cup matches. The control group received instruction on November 24, while the intervention group received training from November 26 to 28, 2022. Both groups completed a demographic questionnaire and pre-test before commencing the class. After the sessions, both groups undertook a post-test immediately. Students were given a CPR scenario as an assignment and asked to outline the BLS steps for this scenario. Subsequently, they completed the SLS-OLE questionnaire, as well as post-tests two weeks and one month after the course.

Finally, educational materials, including AHA guidelines, CPR videos, and info-graphics, were distributed to both groups. Correctly completed assignments were rewarded with gifts, and all participants received a certificate of workshop participation from the nursing school’s scientific association.

### Data analysis

Data analysis involved descriptive statistics (frequency, mean, standard deviation) and analytical statistics. The Shapiro-Wilk test confirmed the assumption of normal data distribution. An independent t-test was employed to compare variances between the two groups, and repeated-measures ANOVA was used to evaluate changes in measurements over time. Data analysis was carried out using SPSS v.23 and MedCalc v.22 software.

### Ethical consideration

Online informed consent was obtained from all participants. The participants were informed that they have no obligation to answer the questionnaire, confidential and anonymous information will be published as a group. Participants could not view the online questionnaire items until they clicked the agree button. All methods were carried out in accordance with relevant guidelines and regulation under the Ethics approval and consent to participate. This study is part of a Master’s degree dissertation and was approved by the ethics committee of the Alborz University of Medical Sciences [Ethic code: IR.ABZUMS.REC.1401.080].

## Results

In this study, 106 nursing students of 120, with a mean age of 21.00 ± 2.1 years, participated. Table 1 presents the demographic and educational information of the nursing students involved in the study.

**Table 1:** The demographic/educational information of the nursing students participating

Variables		*n*	%
Gender	female	54	50.9
	male	52	49.1
academic semester	1	39	36.8
	2	41	38.7
	3	26	24.5
level of interest in field of nursing	high	45	42.5
	moderate	55	51.9
	low	6	5.7
		Mean ± SD (range)	
Age		21 ± 2.1 (18–36)	
GPA(to 20)		16 ± 2.2 (9-19.49)	

**Fig. 2:**
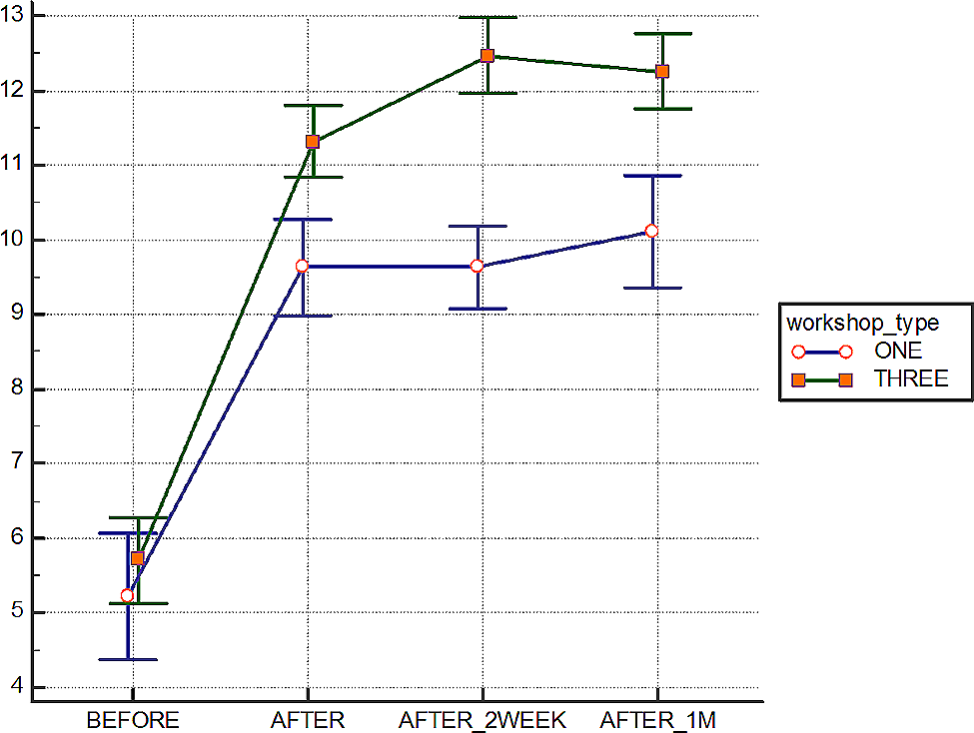
The trend of changing the BLS scores in the different measuring times. X axis: times of assessing BLS score Y axis: BLS score

Comparing the pretest scores between the two groups revealed no significant difference (*p* = 0.35), indicating that the two groups were equivalent in terms of their baseline BLS knowledge. Post-training, knowledge scores improved in both groups, with the intervention group showing a higher increase. Table 2 displays the BLS knowledge scores at different measurement points. Furthermore, the results highlighted that the changes in knowledge scores in both groups were influenced by time (*p* < 0.001), the number of sessions (*p* < 0.001), and their interactive effect (*p* < 0.001) (Fig. 2).

**Table 2:** BLS knowledge scores at different measurement times

Groups	Measurement times									
	Pre test	Post-test immediately	Post-test two week after	Post-test one month after	time		number of sessions		Time* number of sessions	
					sig	Eta squared	sig	Eta squared	sig	Eta squared
Control group	5.22 ± 2.4	9.62 ± 1.9	9.62 ± 1.6	10.11 ± 2.2	< 0.001	0.739	< 0.001	0.30	< 0.001	0.085
Intervention group	5.70 ± 2.0	11.31 ± 1.7	12.47 ± 1.7	12.25 ± 1.7						

Table 3 provides the scores for the SLS-OLE domains in both groups. The results show no statistically significant differences between the two groups. Notably, the highest score was in the perceived learning domain, while the lowest score was in the learner interaction domain.

**Table 3:** Satisfaction scores between groups

Domains of satisfaction scale	Group	min	max	mean	SD	t	sig
Course Structure	Control	12	22	16.46	1.9	1.4-	0.14
	Intervention	11	21	17.31	2.6		
Learner Interaction	Control	11	31	23.31	4	0.97	0.33
	Intervention	17	30	22.50	2.9		
Student Engagement	Control	13	21	17.62	2.1	0.15-	0.88
	Intervention	12	21	17.71	2.5		
Instructor Presence	Control	11	22	17.50	2.5	1.4-	0.15
	Intervention	13	25	18.39	2.6		
Students’ Satisfaction	Control	13	29	22.28	2.8	0.2-	0.84
	Intervention	10	29	22.44	2.9		
Perceived Learning	Control	11	26	23	3.2	0.09-	0.92
	Intervention	10	26	23.07	3.8		

## Discussion

This study aimed to explore the impact of spaced E-learning on Basic Life Support (BLS) knowledge and the satisfaction of nursing students. Spaced learning was chosen as the intervention method, believed to be superior to mass learning [15]. Spaced learning has demonstrated its effectiveness in various fields, including medical sciences [27], mathematics [28], and has been associated with improved knowledge acquisition, retention, skills, competencies, and overall student satisfaction [29, 30] .

The results of this study indicated that BLS training improved in both groups, with the intervention group (spaced learning) showing a greater increase in knowledge, in line with similar studies investigating CPR training. Patocka et al. (2019) compared spaced learning with massed learning in teaching CPR to emergency medical service (EMS) providers, showing improved three-month retention of skills in the spaced learning group [29]. Similarly, in a study by Ferguson et al. (2019), atrial fibrillation training through Mobile spaced education led to higher knowledge and awareness scores among nurses [31]. Beyond CPR, other studies explored the efficacy of spaced learning in various medical fields, such as laparoscopy training [32], microsurgery methods for medical students [33] anesthesia in neurosurgery among anesthesia students [34] and symptom management among nursing students [35]. These studies collectively demonstrate the acceptability and efficacy of mobile distance learning, enhancing both learning outcomes and the ability to apply knowledge in clinical practice.

The not significant increase in BLS knowledge observed in the control group between time 2 and time 3, in contrast to the decrease in knowledge seen in the intervention group during the same period, can be attributed to the spacing effect and the dynamics of memory retention.

The spacing effect, a well-documented cognitive phenomenon, suggests that spreading out learning over time leads to better long-term retention compared to cramming information in a single session. In this study, the control group underwent massed learning in a single three-hour session, likely resulting in a more immediate but less enduring increase in knowledge. On the other hand, the intervention group engaged in spaced learning across three one-hour sessions, allowing for more effective encoding and retention of information over time.

The impact of time and the number of sessions on students’ knowledge scores suggests that knowledge repetition through content review and multiple post-tests enhances retention. Moreover, conducting sessions in multiple segments allows students to better understand the content and seek clarification on unclear topics, as supported by previous research [15, 23]. Longitudinal studies have emphasized the need for repeated CPR training throughout nursing education to maintain knowledge and skills [36]. In this study, spaced learning consisted of three one-hour sessions, with the duration of classes directly influencing learning outcomes. Shorter sessions of 10 to 20 min appear to be more effective, aligning with students’ preferences for shorter, frequent classes over lengthy sessions [37]. This a suggestion for further research to examine the effects of shorter class duration.

Furthermore, the study revealed a high level of student satisfaction with the online course in both groups, with no significant difference between them. Also, Teo et al. study reported no significant differences in satisfaction scores between the two groups of mass and spaced learning in microsurgical procedures [33]. Studies by Dabiri et al. (2019) and Watchmaker et al. (2019) corroborated this finding, highlighting high satisfaction with spaced learning [38, 39]. Reasons for this satisfaction include reduced mental fatigue, enhanced comprehension, more engaging learning experiences, and improved long-term retention [19]. The utilization of various teaching methods and new approaches by instructors also contributes to student satisfaction. After COVID-19 pandemic, the effectiveness of alternative educational methods, such as simulation, educational videos, and games, in teaching clinical topics was highlighted [5].

### Limitation

Data were collected from one center, so it affects the findings’ generalizability. In December, we encountered the challenge of coordinating the training days of our online course with the schedule of the 2022 FIFA World Cup. We devised polls in collaboration and coordination with students to determine the most suitable time for conducting classes in Telegram groups. The final limitation was we measured the BLS knowledge of nursing students, not their skills. So, comparing the knowledge application in the real clinical environment between the two groups is suggested.

### Implications

The findings of this study carry several practical and educational implications for the field of nursing education and beyond:

Enhancing CPR Training: The use of spaced E-learning for CPR training offers a promising avenue for improving the quality and effectiveness of training for nursing students. Implementing this approach in nursing curricula can help ensure that students acquire and retain essential life-saving skills.

Long-Term Retention: The study underscores the importance of regular CPR training sessions throughout the nursing education journey. Institutions should consider incorporating spaced learning approaches to promote long-term retention of knowledge and skills.

Online Education Integration: In the post-COVID-19 era, online education has gained prominence. Nursing schools and institutions should explore the integration of spaced E-learning methodologies to enhance students’ learning experiences, irrespective of whether classes are conducted in person or online.

Effectiveness of Spaced Learning: This study reaffirms the efficacy of spaced learning as a teaching methodology. Educators and instructional designers can adopt this method in various academic disciplines, particularly for complex topics requiring knowledge retention.

Student Satisfaction: The high levels of student satisfaction with spaced E-learning emphasize the importance of using innovative teaching methods that not only facilitate learning but also create engaging and enjoyable educational experiences. Instructors should consider diversifying their teaching strategies.

Use of Technology: The study underscores the role of technology in modern education. Institutions should continue to invest in and promote the use of online platforms and digital resources to support spaced learning initiatives.

Research and Innovation: The study highlights the value of research and innovation in education. Future research can delve deeper into the impact of spaced learning on various topics and different educational contexts, contributing to the advancement of teaching and learning practices. In addition, it is recommended that future studies investigate the effects of spaced learning over different time durations of classes and intervals between sessions; explore differences based on gender, ethnicity, etc.; and focus on skills-based content. The adoption of spaced learning is recommended for face-to-face classes and various theoretical and practical subjects in nursing education.

In conclusion, this study provides valuable insights into the potential benefits of spaced E-learning in nursing education. By considering the implications outlined above, nursing schools, educators, and institutions can embrace innovative approaches to curriculum design, teaching methodologies, and technology integration, ultimately enhancing the quality of education and, most importantly, the quality of patient care.

## Conclusion

CPR training is a critical skill for all students, particularly nursing students. This study found that students who underwent spaced learning exhibited greater knowledge acquisition at various measurement points compared to the control group (mass learning). Interestingly, both groups reported similar levels of satisfaction. Considering the significance and increasing prominence of online education, spaced E-learning emerges as an effective approach for teaching and enhancing knowledge retention among nursing students. Teaching content in multiple sessions proves to be more effective than massed learning in promoting nursing students’ learning and knowledge retention.

## Data Availability

The datasets used and/or analyzed during the current study are available from the corresponding author on reasonable request.

## References

[1] Feazdeesfani H (2019). Assessment of Theoretical Knowledge of Cardio-Pulmonary resuscitation in residents of various specialties in Mashhad universities of Medical sciences. Horizons Med Educ Dev.

[2] Nishara M et al. *Knowledge and attitudes regarding Basic life support among the final yearundergraduates in selected faculties of the University of Colombo (UOC)* 2023.

[3] Cartledge S (2016). A systematic review of basic life support training targeted to family members of high-risk cardiac patients. Resuscitation.

[4] Javaheri Arasteh A, Najafi Ghezeljeh T, Haghani S (2018). Effects of peer-assisted education on the knowledge and performance of nursing students in basic cardiopulmonary resuscitation. Iran J Nurs.

[5] Ali DM (2021). Cardiopulmonary resuscitation (CPR) training strategies in the times of COVID-19: a systematic literature review comparing different training methodologies. Scand J Trauma Resusc Emerg Med.

[6] Yazdani M, Farsi Z, Nezamzadeh M (2018). Cardiopulmonary resuscitation education with serious game on base smart phone and simulation on the attitude of nursing students in Aja University of Medical Sciences. Military Caring Sci J.

[7] Kim E-A, Cho K-J (2023). Comparing the effectiveness of two new CPR training methods in Korea: medical virtual reality simulation and flipped learning. Iran J Public Health.

[8] Saidu A (2023). Effectiveness of video self-instruction training on cardiopulmonary resuscitation retention of knowledge and skills among nurses in north-western Nigeria. Front Public Health.

[9] Khaledi A (2024). Comparison of gamification and role-playing education on nursing students’ cardiopulmonary resuscitation self-efficacy. BMC Med Educ.

[10] Ziabari SMZ et al. Continuous education of basic life support (BLS) through social media; a quasi-experimental study. Archives Acad Emerg Med, 2019. 7(1). PMC6377214 30847439

[11] Demirtas A (2021). Effectiveness of simulation-based cardiopulmonary resuscitation training programs on fourth-year nursing students. Australasian Emerg Care.

[12] Kim JW (2016). Improvement in trainees’ attitude and resuscitation quality with repeated cardiopulmonary resuscitation training: cross-sectional simulation study. Simul Healthc.

[13] Kim SH, Kim SH, Shim CS (2007). The effect and retention of CPR training in nursing students. J Korean Soc Emerg Med.

[14] Sharan S, Tan IGC. Duration of Class Sessions and the Problem of Teaching Method. Organizing Schools for Productive Learning; 2008. pp. 67–75.

[15] Trout B (2018). The effect of class session length on student performance, homework, and instructor evaluations in an introductory accounting course. J Educ Bus.

[16] Alavian F (2017). Distance Learning: creating the Right Space for Neuroscience in the Classroom. J Educ Basic Sci.

[17] Versteeg M (2020). Conceptualising spaced learning in health professions education: a scoping review. Med Educ.

[18] O’Hare L et al. *A pilot randomized controlled trial comparing the effectiveness of different spaced learning models used during school examination revision: the SMART Spaces 24/10 model*. in *Frontiers in Education*. 2023. Frontiers.

[19] Singh H. *What is Spaced Learning & (Why Does it Matter) in eLearning?* 2021 [cited 2022 22.02.2022]; https://www.instancy.com/blog/what-is-spaced-learning/.

[20] Sharifdini M, Evazalipour M, Hesari Z (2023). Virtual spaced-learning method, during COVID-19 for Pharm D students. BMC Med Educ.

[21] Donker SC (2022). Retrieval practice and spaced learning: preventing loss of knowledge in Dutch medical sciences students in an ecologically valid setting. BMC Med Educ.

[22] Yeung J (2020). Spaced learning versus massed learning in resuscitation—a systematic review. Resuscitation.

[23] Oermann MH (2020). Training interval in cardiopulmonary resuscitation. PLoS ONE.

[24] Chung J, Chen H-C (2020). Development and psychometric properties of student perceptions of an online course (SPOC) in an RN-to-BSN program. Nurse Educ Today.

[25] Hoseinzadeh E (2021). Factors affecting nursing student acceptance of online learning under the COVID-19 pandemic. Iran J Med Educ.

[26] Gray JA, DiLoreto M (2016). The effects of student engagement, student satisfaction, and perceived learning in online learning environments. Int J Educational Leadersh Preparation.

[27] Kamali M, Mousavi SK. The effect of spaced learning method on the evaluation score and education quality in nursing students. Med Teach, 2024: p. 1–8. 10.1080/0142159X.2024.2308057 38295521

[28] Hopkins RF (2016). Spaced retrieval practice increases college students’ short-and long-term retention of mathematics knowledge. Educational Psychol Rev.

[29] Patocka C (2019). A randomized education trial of spaced versus massed instruction to improve acquisition and retention of paediatric resuscitation skills in emergency medical service (EMS) providers. Resuscitation.

[30] Smeds MR (2016). Mobile spaced education for surgery rotation improves National Board of Medical Examiners scores. J Surg Res.

[31] Ferguson C (2019). An mHealth intervention to improve nurses’ atrial fibrillation and anticoagulation knowledge and practice: the EVICOAG study. Eur J Cardiovasc Nurs.

[32] Boettcher M (2018). The spaced learning concept significantly improves training for laparoscopic suturing: a pilot randomized controlled study. Surg Endosc.

[33] Teo WZ (2021). Randomized controlled trial comparing the effectiveness of mass and spaced learning in microsurgical procedures using computer aided assessment. Sci Rep.

[34] Khalafi A, Fallah Z, Sharif-Nia H. The effect of spaced learning on the learning outcome and retention of nurse anesthesia students: a randomized-controlled study. BMC Med Educ. 2024;24(1). 10.1186/s12909-024-05290-9 10.1186/s12909-024-05290-9 PMC10958887 38515084

[35] Mc Veigh C (2022). Pilot study to explore the use of mobile spaced learning as a digital learning platform when teaching symptom management to undergraduate nursing students: SPLENdidS study. PLoS ONE.

[36] Adib-Hajbaghery M, Azizi-Fini E (2013). Longitudinal study of cardiopulmonary resuscitation knowledge and skills among nurse interns of Kashan university of medical sciences. Iran J Med Educ.

[37] Zha S, Estes MD, Xu L (2019). A Meta-analysis on the Effect of Duration, Task, and training in Peer-Led learning. J Peer Learn.

[38] Dabiri S, Mohammadi A, Mojtahedzadeh R (2019). The effect of test-enhanced spaced learning on the otolaryngology board and annual examination results: a quasi-experimental study. J Adv Med Educ Professionalism.

[39] Watchmaker J, Gonzales EC, Larson AR. Interactive teaching and repeat exposure maximize medical student satisfaction but do not promote long-term retention of dermatologic knowledge. Dermatol Online J, 2019. 25(9). 31738839

